# The Role of Fetal MRI in the Evaluation of Ventriculomegaly: A Scoping Review †

**DOI:** 10.3390/children13070971

**Published:** 2026-07-22

**Authors:** Annabella Lewis, Nadia Badawi, Rod Hunt, Esther Tantsis, James Christie, Hannah Dalrymple

**Affiliations:** 1 Sydney Medical School, The University of Sydney, Camperdown, NSW 2050, Australia; 2 Cerebral Palsy Alliance, The Children’s Hospital at Westmead, The University of Sydney, Camperdown, NSW 2050, Australia; esther.tantsis@health.nsw.gov.au; 3 Monash Health, Cerebral Palsy Alliance, Monash University, Clayton, VIC 3800, Australia; 4 The Royal Australian and New Zealand College of Radiologists, The Children’s Hospital at Westmead, The University of Sydney, Camperdown, NSW 2050, Australia; 5 The Children’s Hospital at Westmead, The University of Sydney, Camperdown, NSW 2050, Australia

**Keywords:** fetal MRI, neurodevelopmental outcomes, prenatal counseling, review, ventriculomegaly

## Abstract

**Highlights:**

**What are the main findings?**

- Fetal MRI yielded additional or different imaging findings to ultrasound (60.1%). It was equivalent to postnatal MRI in 47.2%.
- Neurodevelopmental issues after fetal ventriculomegaly were common (63.5%), especially with additional brain anomalies.

**What are the implications of the main findings?**

- Fetal MRI may contribute to prognostication and counseling in pregnancies affected by ventriculomegaly.
- As fields like clinical genetics and prenatal surgery develop alongside fetal MRI, its role is likely to evolve.

**Abstract:**

**Background/Objectives**: Fetal MRI is used increasingly to detect fetal brain anomalies despite limited high-quality evidence. The common anomaly, ventriculomegaly, exemplifies the challenge of using fetal MRI for neurodevelopmental prognostication. **Methods**: This scoping review followed JBI guidelines. Four electronic databases were searched without restriction. Screened studies were eligible where they included primary data with fetal MRI showing ventriculomegaly and clinical investigations, management, or fetal outcomes. A descriptive numerical summary with data-driven bivariate and multivariate analysis of case-level data was undertaken. **Results**: A total of 345 studies were included (1027 cases, 121 cohorts). Fetal MRI was performed at a mean of 28 weeks’ gestation. It yielded additional or different imaging findings to US in 60.1%. Fetal results differed from neonatal MRI in 52.8%. Discrepancy between imaging modalities has increased from 1986–2025. About 69.1% of fetuses were liveborn, 28.9% were terminated and 1.9% died in utero. Postnatal neurosurgery was common after severe ventriculomegaly (36.7%). Neurodevelopmental issues were common (63.5%), especially where additional brain anomalies were detected on fetal MRI (OR 6.40 (95% CI 4.02–10.2), *p* < 0.001). **Conclusions**: Fetal MRI may contribute to neurodevelopmental prognostication in fetal ventriculomegaly in conjunction with other investigations. Available evidence is of mixed quality with variable reporting, relying on retrospective studies and non-standardized outcome measures. Long-term prospective studies are required.

## 1. Introduction

Fetal brain anomalies cause many common neurodevelopmental disorders [1,2]. Fetal MRI emerged in the 1980s as a potentially sensitive, specific tool to identify subtle anomalies in at-risk patients [3,4,5]. Clinical interest has exploded [3,6]. Fetal anomaly detection has been linked to higher rates of pregnancy termination (TOP) and neurodevelopmental disorders such as cerebral palsy [2,7,8,9,10]. Despite the dissemination of a large volume of individual retrospective reports, little high-quality evidence exists to guide fetal MRI interpretation, particularly with regards to neurodevelopmental outcome [11,12]. Nonetheless, it is increasingly utilized in obstetric practice to counsel patients around pregnancy and postnatal care decisions including TOP. Our study summarizes the current state of the evidence and highlights areas of uncertainty relevant to perinatal counseling.

Diagnostic accuracy compares favorably to ultrasound (US) [4,13,14,15]; though fetal MRI can add clinical information [13,14,15,16]. Van Doorn [14] reported 65% agreement between fetal MRI and US, and Jarvis [15] reported additional findings in 16.5% with the same primary diagnosis. Concerns about interpretation [17] and importance relative to genetic and infectious disease testing qualify its utility [3,18]. Therefore, meaningfully translating fetal MRI findings to inform management and prognosis remains challenging [3].

Estimates of diagnostic accuracy compared to postnatal imaging and outcome vary widely [14]. Arechvo [19] reported 73.8% confirmation of fetal findings. The “MERIDIAN” study [4] reported a promising 93%; however, 42% of infants with neurodevelopmental issues had normal outcomes predicted by fetal MRI [4]. Given accuracy can be affected by gestational age (GA), technique, radiologist expertise, and disease pathogenesis [5,19], real-world reliability is unclear, especially in predicting neurodevelopmental outcome [3].

The most common indication for fetal MRI is ventriculomegaly (VM) [6,20,21]. VM is a heterogeneous condition affecting approximately 1% of fetuses and typifies challenges of prenatal counseling [22]. Comprehensive evaluation of neuroanatomy, genetics, and infection are important since findings influence prognosis [6,20,23,24,25,26,27]. Severity of VM on US is linked to neurodevelopmental outcome [24,25,28,29] and postnatal neurosurgical intervention [30,31], particularly for spinal dysraphism [32,33]. Whether MRI and the additional depth of neuroanatomical detail it can reveal has a role in prognostication independent of other investigations remains controversial. While a 2019 systematic review [34] showed MRI had minimal additional utility compared to high-quality neurosonography for fetal VM, MERIDIAN subgroup analysis [35] revealed an independent effect on management in 51.7%.

Fetal MRI continues to generate enthusiasm. Urgent research is required to enable informed, shared decision-making between families and clinicians [4,24,31]. We aimed to investigate how fetal MRI influences the investigation, management, and prognostic outcomes for fetuses with VM.

## 2. Materials and Methods

A scoping review was conducted utilizing the JBI [36] and Arksey and O’Malley frameworks [37] following PRISMA guidelines. A scoping review was conducted given their utility to summarize large quantities of emerging evidence and identify knowledge gaps. Patient consent statement is not applicable or needed for this study.

We searched the databases Medline, PubMed, Embase, and Maternity and Infant Care (18 May 2025) without any restrictions. Search terms included: ((Brain AND Abnormalities, Multiple) OR ventricular adj3 abnormalit* OR ventriculomegaly) AND (Fetus OR Pregnancy or f?etus OR f?etal OR pregnanc* OR in utero) AND (Magnetic Resonance Imaging or magnetic resonance imag* OR MRI) (see Supplementary Material).

Included studies reported human fetuses with VM undergoing MRI; at least one of investigations, management or outcomes; and were published in English. Exclusion criteria were reviews, abstracts, posters, or conference proceedings, and articles discussing imaging without clinical context.

Papers were screened independently by two reviewers for inclusion with conflicts resolved by discussion. Duplicate patients were identified by cross-referencing study centers, clinical settings, authors, and patient characteristics to avoid double counting. Data was extracted as individual case data using a REDCap form [38], or as collated cohort data if insufficient detail was published.

An exploratory data analysis was undertaken in IBM SPSS Statistics 29. For cases, data-driven analysis was used including post hoc analysis with the Bonferroni correction. Two-sided bivariate analysis (Chi-squared, two-sample independent t, ANOVA, and Mann–Whitney U tests) and multivariate logistic regression adjusting for study type and year of publication were used. Regression assumptions were checked using *t*-testing. Missing data was not imputed with cases omitted from the relevant analyses as required. For all analyses, a *p*-value <0.05 was considered significant. In keeping with the purpose of a scoping review, the analysis is intended to map and summarize the available literature; results reflect associations in the literature and may not reflect true causation.

Where VM severity was unspecified measurements were coded using Society for Maternal-Fetal Medicine (SMFM) Guidelines [11]. Imaging was “concordant” if all aspects of reported findings were compatible (there were no missing findings nor additional findings apart from direct, local evolution arising from changes at earlier timepoints).

This scoping review is registered with Open Science Framework (registered 11 October 2025, registration number 10.17605/OSF.IO/RXZ7K, https://doi.org/10.17605/OSF.IO/RXZ7K).

## 3. Results

### 3.1. Evidence Sources and Study Characteristics

**Figure 1:**
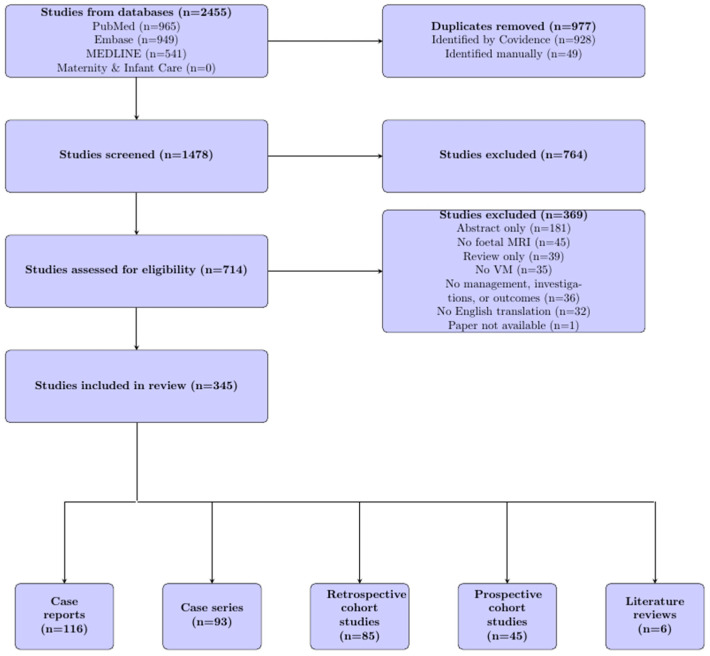
PRISMA flow diagram of included and excluded studies with breakdown of included study types. Studies were published in 40 countries, with the largest number published in the USA (*n* = 100), followed by France (*n* = 44), Italy (*n* = 39), China (*n* = 27), Israel (*n* = 25), and the UK (*n* = 24).

The search returned 1478 studies, of which 345 were included (Figure 1). Included studies were published from April 1986 onwards and reported 1027 individual cases and 121 cohorts (7192 fetuses in total with VM on fetal MRI). Overall, 294 studies were retrospective and 46 prospective. Patient demographics were inconsistently reported (Table 1).

### 3.2. Fetal MRI Findings

Where reported, fetal MRIs were performed using 1.5T scanners using a T2 sequence. Mean GA for cases at an average of 28.3 weeks (SD 5.17, range 14 to 42 weeks) (Table 2) did not change over years of publication (*p* = 0.06). However, an increasing proportion over time (OR 1.06, *p* < 0.01) did not report GA at MRI (11.9% overall).

Table 2 presents the features of fetal MRI for individual cases. VM was most commonly mild; however, over time, studies have reported greater severity (OR 1.05, *p* < 0.01). Despite studies increasingly classifying the VM severity (OR 1.03, *p* < 0.01), definition used was variable: only 46 utilized SMFM guidelines [11]; 247 studies did not publish the definition used. Brain anomalies with VM were more common than isolated VM and are more commonly reported over time (OR 1.05, *p* < 0.01).

There was a 60% discordance between findings on fetal MRI and US, increasing over time in published studies (OR 1.04, *p* < 0.01), despite reduced likelihood of publishing sufficient detail to ascertain concordance over time (OR 1.05, *p* < 0.01).

A total of 34 cases and seven cohorts reported subsequent MRI in the fetal period. This was usually performed following in utero spinal dysraphism repair (five cohorts, four cases) or other invasive procedures [39,40,41,42,43,44]. Indication was unknown for 29 cases.

### 3.3. Other Investigations

#### 3.3.1. Genetic Investigations

**Figure 2:**
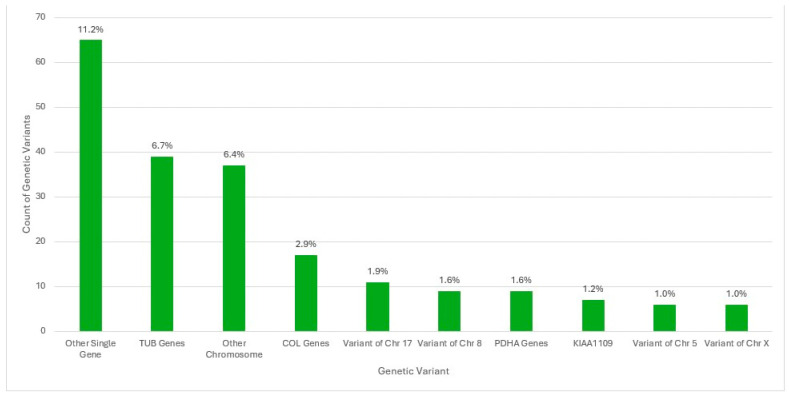
Genetic variants reported of fetal VM on MRI (percentages are for *n* = 579 cases for which the results of genetic testing were reported).

Case Data: A total of 592 cases underwent genetic testing (Table 3), with likelihood of testing increasing over time (OR 1.03, *p* < 0.01). Only 137 had genetic counseling. Approximately one third had pathogenic or likely pathogenic variants detected (Figure 2), which were reported more commonly over time (OR 1.12, *p* < 0.01 adjusted).

Cohort Data: A normal genetic analysis was an inclusion criterion for 23 cohorts. Karyotype was the commonest genetic investigation, with its use reported in 4321 fetuses in 63 cohorts. Chromosomal microarray was reported in 771 fetuses in 34 cohorts. Other genetic tests were less common. Timing of testing varied: karyotype and microarray were commonly antenatal; gene panels were largely postnatal.

#### 3.3.2. Congenital Infections

Case Data: Table 3 displays the rates of detected prenatal infection. Infective causes were detected infrequently, including cytomegalovirus (*n* = 40), zika virus (*n* = 26) [45,46,47,48,49,50,51], and other TORCH or viral infections (*n* = 6). Infection detection has increased over time (OR 1.10, *p* < 0.01) with a relatively constant rate of testing (*p* = 0.63).

Cohort Data: A total of 66 cohorts tested for TORCH infections with a negative screen required in 23 studies. Fetuses were positive within cohorts for CMV (13 cohorts), zika virus (two cohorts) [52,53], and other TORCH or viral infections (*n* = 9). A total of 13 cohorts reported testing for infection but did not specify results.

### 3.4. Pregnancy Management and Outcomes

#### 3.4.1. Prenatal Interventions

Case Data: A total of 39 fetuses underwent prenatal intervention following MRI including CSF shunting (*n* = 10), spinal dysraphism repair (*n* = 8), management of twin-twin transfusion syndrome (*n* = 6), amnioreduction (*n* = 3) and vesico-amniotic shunting (*n* = 1). Medical interventions were for anemia or coagulopathy (*n* = 5) [54,55,56], treatment of congenital infection (*n* = 3) and SVT (*n* = 1).

Cohort Data: A total of 13 cohorts reported prenatal intervention (spinal dysraphism repair (*n* = 10), intervention for TTTS (*n* = 2), and intrauterine transfusion (*n* = 1) [57]).

#### 3.4.2. Pregnancy Outcomes

Case Data: A total of 964 cases reported pregnancy outcome: 667 cases of live birth, 279 (28.9%) termination, and 18 (1.87%) fetal demise (Table 2). There has been no significant change in the live birth rate over time (*p* = 0.33); however, studies have become more likely not to report pregnancy outcome (OR 0.97, *p* = 0.06). The median GA at delivery was 37 weeks (IQR 34, 38, *n* = 401). Fetal MRI influenced the birth plan in 135 cases, and postnatal management in 74.

Postnatal mortality was high at 11.2%, including 51 neonatal deaths. Mean birthweight was 2.67 kg (SD = 0.780, *n* = 168) with median length of hospital stay of 3 days (IQR 0, 27, *n* = 48). Autopsy results were infrequently reported; however, discordance with fetal MRI was common with 20 detecting additional findings, five failing to find VM, one failing to identify a mass lesion, and five affected by fetal autolysis.

Live birth was more likely in fetuses with isolated VM compared to fetuses with additional brain anomalies (*p* < 0.001, OR 0.301 (95% CI 0.196, 0.460)). Pregnancy survival was more common in severe VM cases (*p* = 0.03).

Live birth was less common in fetuses that underwent earlier MRI (*p* < 0.001). Mean GA was 29.17 weeks (SD = 4.980) for live births compared with 26.6 (SD = 5.201) in non-survivors. This association has diminished over time (*p* < 0.01). The effect of GA on TOP (*p* < 0.01), not fetal demise (*p* = 0.88), accounted for this.

### 3.5. Postnatal Imaging

Case Data: A total of 335 neonates underwent MRI, 132 transcranial US, and 50 CT. A total of 177 (52.8%) neonatal MRIs gave results that differed to the fetal MRI (Table 2): 140 yielded new findings and 21 (6.3%) failed to demonstrate CNS findings detected on fetal MRI. A total of 16 neonatal MRIs differed only in VM severity. A total of 231 (78.0%) neonatal MRIs were concordant with fetal MRI for VM severity: 15 cases showed VM resolution and 23 progression. Fetal and neonatal MRI discordance has not changed significantly over time (*p* = 0.93). There was no relationship between GA at fetal MRI and agreement with postnatal MRI (*p* = 0.23). Mean GA was 29.7 weeks (SD 5.18) in concordant MRI and 28.9 (SD = 5.15) in discordant.

Cohort Data: A total of 53 cohorts reported performing neonatal MRI, 29 transcranial US, and seven CT. A total of 32 cohorts reported neonatal MRI concordance with fetal MRI VM severity in at least some fetuses. A total of 29 cohorts reported that neonatal MRI revealed additional findings in some cases, compared to 13 in which neonatal MRI failed to elucidate prenatal findings in some fetuses.

### 3.6. Postnatal Neurosurgical Intervention

Case Data: A total of 120 (18.0%) fetuses required at least one postnatal neurosurgical intervention. The commonest interventions were ventricular shunt (*n* = 108) or reservoir (*n* = 5), ventriculostomy (*n* = 13), cyst or ventricular fenestration (*n* = 5), and choroid plexus cauterization (*n* = 5). Intervention rates have not changed significantly over time (*p* = 0.28).

There was a significant association between VM severity and postnatal neurosurgical intervention (*p* < 0.01), with severe VM and larger ventricular diameters more likely to undergo intervention (Table A1). There was no difference in the need for neurosurgical intervention in isolated or non-isolated VM (*p* = 0.439, OR 0.833 (95% CI 0.524, 1.325)).

Cohort Data: A total of 42 cohorts reported postnatal neurosurgical intervention was indicated for at least one fetus. Most commonly, these were for CSF diversion (37 cohorts) or spinal dysraphism repair (nine cohorts).

### 3.7. Neurodevelopmental Outcomes

Only 45 studies employed validated tools to measure neurodevelopmental outcomes. The most used scales were: Bayley Scales of Infant and Toddler Development [58] (*n* = 14); Ages and Stages Questionnaires [59] (*n* = 9); Denver Developmental Screening Tests [60] (*n* = 6); and Wechsler Preschool and Primary Scale of Intelligence [61] (*n* = 5). Age at follow-up varied significantly (median 14 months, IQR 6,36).

**Figure 3:**
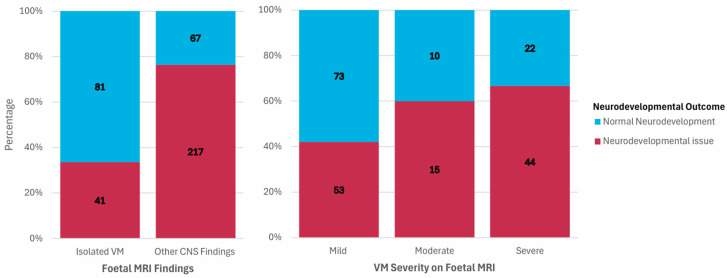
Association between fetal MRI findings and the presence of a neurodevelopmental issue on Chi-squared or Fisher’s exact test by study type. * Significant for *p* < 0.05. ** Significant for *p* < 0.001. Additional brain anomalies: ** Prospective cohort studies: χ^2^ = 42.51, df = 1, *p* < 0.001, OR = 21.79 (95% CI 7.61, 62.35). ** Retrospective cohort studies: χ^2^ = 12.52, df = 1, *p* < 0.001, OR = 4.71 (95% CI 1.93, 11.49). ** Case series: χ^2^ = 20.28, df = 1, *p* < 0.001, OR = 6.33 (95% CI 2.71, 14.80). Case reports: Fisher’s exact test *p* = 1.00, OR = 0.55 (95% CI 0.06, 5.03). ** Overall: χ^2^ = 67.49, df = 1, *p* < 0.001, OR = 6.40 (95% CI 4.02, 10.19). There was also one case drawn from review figures whose data is not represented in this figure. VM severity: Prospective cohort studies: insufficient data. Retrospective cohort studies: χ^2^ = 4.99, df = 2, *p* = 0.082. Case series: insufficient data. Case reports: insufficient data. * Overall: χ^2^ =11.29, df = 2, *p* = 0.004 (*p* = 0.02 adjusted).

Case Data: Neurodevelopmental outcome was reported in 408 cases, and increasingly in more recent cohorts (OR 1.02, *p* = 0.04). Neurodevelopment was normal in 149 (36.5%). Detailed reporting was limited; motor impairments (*n* = 117) and seizures (*n* = 81) were the commonest issues specified. An abnormal neurodevelopmental outcome was significantly more likely in fetuses with VM and additional brain anomalies on MRI (OR 6.399 (95% CI 4.019, 10.186)) (Figure 3). Neurodevelopmental issues were associated with more severe fetal VM (Figure 3, Table A1).

Cohort Data: Neurodevelopmental outcome was reported in 65 cohorts and most included infants with normal (42 cohorts) and abnormal (58 cohorts) outcomes. Specific issues included motor (36 cohorts), cognitive or language (19 cohorts), vision (nine cohorts) and hearing (two cohorts) impairments, and seizures (16 cohorts).

## 4. Discussion

The body of evidence around fetal MRI is growing; to our knowledge, our study is the first comprehensive summary. Fetal MRI yielded additional or different findings to US in 60.1%. Despite low concordance with postnatal MRI, it is associated with neurosurgical interventions in severe VM and poorer neurodevelopmental outcomes for fetuses with additional brain anomalies. This exploratory review found that fetal MRI may be a valuable adjunct to neurodevelopmental counseling in fetal VM.

In our review, fetal MRI yielded additional or different findings to US in 55.9–68.4% of cases. Previous systematic reviews reported less difference, ranging from 27.5% [16] to 40% [13,14,15]. Two reasons for this discrepancy seem likely: firstly, discordance was only reported for new pathology or management [13,14,15,16]; secondly, these works included all fetal MRIs, while this review considered only VM, where MRI actively seeks differences to US [20]. Further, this review included scans performed with a variable level of radiologist experience, a complicating factor reflected in real-world practice. The clinical relevance of subtle differences between MRI and US, common in this review, is unclear and was poorly reported in the literature.

Diagnostic accuracy is uncertain. In our review, 52.8% of fetal MRIs were non-equivalent to postnatal MRI and 31 autopsies were discordant. This may reflect the evolution of pathology over time, highlighting the importance of longitudinal assessment [62]. Concordance rates have not increased over time, possibly due to increased TOP, improved fetal MRI resolution to detect subtle findings [5], concurrent postnatal MRI improvements, or publication bias. In other studies, concordance varied from 69–93% [62,63]. Such variation may reflect different study populations and definitions of “accuracy”. Importantly, in our review, most discrepancy (79.2%) was due to neonatal MRI identifying additional anomalies. This was much more than the MERIDIAN study [63] VM subgroup (10%) but parallels results by Gumayan [62]. Additional anomalies detected on neonatal MRI may have arisen at later GAs or postnatally with continued growth and cortical maturation [62].

Fetal MRI is usually recommended in the second or third trimester, but optimal GA is little studied [12,64,65]. This is significant given legal and sociocultural implications of TOP. Our review revealed marked variability in the effect of GA. In retrospective cohorts, earlier fetal MRIs were more likely to differ from neonatal MRI. GA was associated with VM severity for prospective cohorts and overall. This could reflect evolving pathology, although SMFM guidelines suggest only 16% of VM progresses [11]. Caution is required pending further research as earlier imaging may not reflect emergent neurodevelopmental pathophysiology.

Adverse neurodevelopmental outcomes were common after fetal VM, particularly in those with additional brain anomalies. Only case reports differed, likely due to small sample size (54). Our findings align with older non-MRI-based studies where neurodevelopmental issues affected up to 91% with additional anomalies [25,26,27,28]. More severe VM trended towards adverse neurodevelopmental outcomes, similar to Ali’s [29] recent systematic review of isolated fetal VM. As many fetal MRIs were non-equivalent to US, it appears fetal MRI provides additional information to assist clinicians to discuss neurodevelopmental outcomes with parents. In fact, our review showed that the discrepancy between US and MRI findings has significantly increased over years of publication. Although this may reflect improving resolution and publication and referral bias towards discrepant cases, it also suggests that despite ongoing improvements in neurosonography [34], fetal MRI retains an important clinical role in cases where prognosis remains uncertain based on US findings alone. However, our review is not intended to show causative relationships between fetal MRI findings and neurodevelopmental prognosis. It also includes heterogeneous studies spanning 40 years of technological evolution. Thus, updated systematic reviews to compare fetal MRI and modern neurosonography are warranted. This is vital for parents to make informed decisions.

In our review, live birth occurred in 69.1% of cases with fetal demise rare, compatible with previous estimates for fetal VM [23,25,66]. About 28.9% of pregnancies were terminated. This figure is difficult to extrapolate given legal and bias-related factors creating underreporting, particularly as the literature has become less likely to report pregnancy outcome over time [67]. Changing sociolegal climate may explain our finding that GA affected TOP rates less over time. Strikingly, we found that TOP was more likely in milder VM, but care must be taken interpreting this finding as publication bias towards unusual clinical courses is likely. Publication bias, particularly concerning case studies, is a concern in the literature and limits conclusions to be drawn from this review. Nonetheless, survival was common after severe VM although more likely to require postnatal neurosurgical intervention [68].

Genetic testing was common with fetal MRI. Our 9.8–16.6% yield for different cohorts is compatible with estimates for fetuses with CNS anomalies [69,70]. However, cautious interpretation is needed given genetic technology has evolved considerably over the epoch examined. Clinicians should utilize genetic testing to improve prognostication. However, in those cases without pathogenic or likely pathogenic variants, fetal MRI may be a valuable adjunct investigation. CMV and zika virus were the commonest infections; however, zika testing is not universally indicated outside tropical epidemics [45,46,47,48,49,50,51]. Increasing infection detection may be due to advancements in PCR testing. Further, this review revealed the emerging use of serial fetal MRI for prenatal surgery [39,40,41,42,43,71].

Including many studies and case data was advantageous to our review, allowing stratification by study type and minimizing the limitation of not performing quality appraisal. Conclusions are limited by not completing a meta-analysis or clustering analysis; however, this aligns with the purpose of a scoping review to provide an exploratory rather than causation-focused analysis. As not all studies published individual case data; these could not be included in case-level analysis. We did not search the grey literature or use citation chaining, potentially increasing publication bias impact and causing omission of some eligible studies. Further, included studies were limited to English language, potentially introducing language bias. Similarly, demographic bias is of concern. Despite these limitations, our broad search strategy supported a comprehensive review.

This identified essential problems in the literature, barriers to generalizing our findings and opportunities for future primary studies. Firstly, the literature relies on observational studies. About 87% were retrospective and MERIDIAN [4] is the only large, ongoing prospective study. Risk of bias is high, especially for small studies where TOP may be underreported and neurodevelopmental issues overreported due to ascertainment bias with specialist referral [67]. Future prospective studies or compulsory MRI registries utilizing up-to-date technologies to assess optimal GA and neurodevelopmental outcome may assist clinicians. These studies should also address the emerging use of machine learning [68]. Secondly, many studies failed to report key clinical characteristics and outcomes. Crucially, 60.3% did not report neurodevelopmental outcome. Comprehensive reporting is vital for future multivariate analysis. Thirdly, neurodevelopmental outcomes assessment was inconsistent and prone to measurement bias. Only 13% of studies used a standardized measure, and age at review varied. Similar issues arose around defining VM severity. To capture the breadth of literature, we used “neurodevelopmental issue” to describe a spectrum of ages and outcomes from very mild to severe and life-limiting. This incurs significant heterogeneity in the population examined and our findings regarding the severity and nature of neurodevelopmental outcomes following abnormalities detected on fetal MRI should be approached with caution. Detailed prognostication warrants large, long-term follow-up studies, which can account for the potential modulating effects of different disease processes on the prognostic utility of fetal MRI, particularly as the importance of subtle fetal MRI anomalies remains unquantified and neurodevelopmental disorders evolve over time [1]. This is significant particularly as sample sizes for the various different etiologies of VM in the literature were most often insufficient to facilitate meaningful subgroup analyses. For now, clinicians should advise families that outcomes vary widely for fetal VM on MRI.

## 5. Conclusions

This exploratory, scoping review suggests a prognostic role for fetal MRI. It may be utilized to evaluate fetal VM alongside other tools such as genetic testing. Fetal MRI yielded different findings to US in cases of fetal VM. Additional brain anomalies were associated with adverse neurodevelopmental outcomes. Together these features suggest fetal MRI may aid clinicians to counsel families amidst the uncertainty of prenatal diagnosis. As fields like clinical genetics and prenatal surgery develop alongside fetal MRI, its role is likely to evolve. Open questions remain, particularly around optimal timing for fetal MRI and its capacity for detailed neurodevelopmental prognostication. Future prospective studies with detailed long-term follow-up are needed to clarify its role as imaging techniques and fields like genetics continue to develop. Our review suggests that fetal MRI may be used judiciously as part of counseling in pregnancies affected by VM.

This article is a revised and expanded version of a poster presentation entitled Ventriculomegaly on Foetal MRI–What Does It Mean? which was presented at PSANZ 2026 Congress, 22–25 March 2026 Perth (Noongar Country), Western Australia [72].

## Figures and Tables

**Table 1:** Summary of patient demographics, where reported, for 1027 cases.

Characteristics	Cases
Maternal age (*n* (%))	341
(mean (SD))	30.3 (5.2)
Parity (*n* (%))	201
Primiparous	97 (48.3)
Multiparous	104 (51.7)
Consanguinity (*n* (%))	83
Non-consanguineous	73 (88)
Consanguineous	10 (12)
Parental history (*n* (%))	174
No medical issues	92 (52.9)
Parental neurological issue	13 (7.5)
Other medical issue	15 (8.6)
Twin pregnancy (*n* (%))	1027
Singleton	996 (97.0)
Twin	31 (3.0)
Fetal sex (*n* (%))	385
Female	184 (47.8)
Male	201 (52.2)
Obstetric issue current pregnancy (*n* (%))	107
None	59 (55.1)
Issue	48 (44.9)
Premature rupture of membranes	12
Unequal placental sharing	20
Placental complications	4
Preeclampsia	3
Other	7

**Table 2:** Characteristics of fetal MRI for individual cases of fetal VM with pregnancy outcome sorted by study type from which the case was derived.

	Prospective Cohort Studies (*n* = 232)	Retrospective Cohort Studies (*n* = 254)	Case Series (*n* = 402)	Case Reports (*n* = 121)	All Study Types (*n* = 1027)
Gestational age at fetal MRI					
Weeks mean (SD)	26.8 (5.9)	29.6 (4.3)	28.7 (4.9)	27.7 (5.2)	28.3 (5.2)
*n*	214	191	378	115	905
Severity of VM on MRI *n*	113	167	211	58	551
Mild %	65.5	63.5	51.2	50.0	57.5
Moderate %	12.4	9.0	12.8	15.5	11.8
Severe %	25.0	27.5	36.0	34.5	30.7
MRI brain findings *n*	228	253	397	120	1016
Isolated VM %	27.2	29.0	16.1	14.0	21.4
Aqueductal stenosis %	0.9	2.8	3.8	1.7	2.8
Callosal or CSP anomaly %	24.1	13.0	6.3	5.8	12.0
Posterior fossa anomaly %	3.9	8.7	11.1	15.0	9.4
Supratentorial anomaly %	9.6	17.4	15.4	26.7	16.2
Hemorrhage %	4.4	10.7	8.0	7.5	7.7
Multiple anomalies %	29.8	18.6	39.3	29.2	30.6
Spinal dysraphism *n*	232	254	402	121	1027
Present %	3.0	4.3	6.2	1.7	4.5
MRI vs. ultrasound *n*	222	228	357	114	935
Different %	55.9	61.8	58.5	68.4	60.1
Fetal vs. neonatal MRI *n*	89	51	143	43	335
Different %	57.3	43.1	47.6	76.7	52.8
Pregnancy outcome *n*	203	245	382	118	964
Live birth %	68.5	72.2	67.5	65.3	69.1
Mild *n*	41	66	64	13	184
Moderate *n*	9	12	13	7	41
Severe *n*	19	39	48	14	122
Isolated VM *n*	49	62	49	11	172
Other brain anomalies *n*	90	114	208	66	493
TOP %	29.6	24.9	31.7	31.4	28.9
Mild *n*	21	35	33	15	104
Moderate *n*	5	3	13	0	21
Severe *n*	3	4	23	6	36
Isolated VM *n*	7	7	6	4	24
Other brain anomalies *n*	53	54	114	32	253
Fetal demise %	2.0	2.9	0.8	3.4	1.9
Mild *n*	2	2	2	0	6
Moderate *n*	0	0	0	1	1
Severe *n*	1	2	1	0	4
Isolated VM *n*	2	2	0	0	4
Other brain anomalies *n*	2	5	3	4	14

Lateral ventricle atrial diameter (LVAD). Termination of pregnancy (TOP). There were also 18 cases drawn from review figures whose data is not represented in this table other than as being included in “all study types”. Supratentorial anomalies included: polymicrogyria (*n* = 52); cortical heterotopia (*n* = 51); other abnormal sulcal development (*n* = 45); interhemispheric cyst (*n* = 34); schizencephaly (*n* = 17); lissencephaly (*n* = 15). Posterior fossa anomalies included: abnormalities of the cerebellar vermis (*n* = 54); cerebellar hypoplasia (*n* = 53); Dandy–Walker malformation (*n* = 29); Chiari malformation (*n* = 25); brainstem kinking (*n* = 16).

**Table 3:** Prenatal investigations performed (and their results) undertaken for fetuses that underwent MRI for VM.

		Prospective Cohort Studies (*n* = 232)	Retrospective Cohort Studies (*n* = 254)	Case Series (*n* = 402)	Case Reports (*n* = 121)	All Study Types (*n* = 1027)
Genetic test performed *n*		156	134	212	88	592
	Karyotype *n*	150	122	124	57	454
	Microarray *n*	7	28	35	38	108
	WES/WGS *n*	1	12	78	29	121
	Gene panel *n*	0	10	28	19	57
Genetic test results *n*		145	123	207	89	579
	P/LP variant %	16.6	9.8	46.4	59.6	33.2
	VOUS %	0.0	0.0	1.4	4.5	1.2
	No variant %	83.4	90.2	52.3	36.0	65.6
Infectious disease testing performed *n*		64	124	125	40	354
	Positive %	20.3	16.1	28.0	15.0	21.2
	Negative %	79.7	83.9	72.0	85.0	78.8
Fetal hematology workup performed *n*		1	16	17	20	54
	Anomaly detected *n*	1	16	5	6	28
	Normal results *n*	0	0	12	14	26

Whole exome/genome sequencing (WES/WGS); pathogenic/likely pathogenic (P/LP); variant of uncertain significance (VOUS).

## Data Availability

The data presented in this study are available on request from the corresponding author. The data are not publicly available due to ethical restrictions.

## Supplementary Materials

The following supporting information can be downloaded at: https://www.mdpi.com/article/10.3390/children13070971/s1, the role of fetal MRI in the evaluation of ventriculomegaly: a scoping review search strategy.

## Abbreviations

The following abbreviations are used in this manuscript:

VM	Ventriculomegaly
US	Ultrasound
MRI	Magnetic resonance imaging

## Appendix A

**Table A1:** Subgroup analysis by study type of the effect of ventricular diameter measured on fetal MRI assessed by the Mann–Whitney U test.

	Prospective Cohorts			Retrospective Cohorts			Case Series			Case Reports		
	Diameter Median (IQR)	*n*	*p*	Diameter Median (IQR)	*n*	*p*	Diameter Median (IQR)	*n*	*p*	Diameter Median (IQR)	*n*	*p*
Pregnancy outcome			0.028 *			0.14			0.12			0.14
Live birth	12.2 (11.0, 16.2)	40		13.3 (11.0, 24.3)	46		13.0 (10.7, 17.3)	81		14.0 (12.0, 17.0)	20	
TOP, FDIU, or stillbirth	11.0 (10.0, 13.0)	23		11.8 (10.1, 14.6)	6		14.5 (12.0, 17.0)	35		11.9 (11.0, 14.3)	14	
Postnatal neurosurgery			0.009 *			<0.001 *			0.001 *			0.046 *
Not performed	12.0 (11.0, 14.0)	35		12.0 (10.8, 22.0)	35		11.0 (10.2, 13.2)	53		13.0 (11.0, 16.3)	13	
Performed	18.0 (15.0, 24.0)	5		26.3 (14.4, 29.4)	11		18.3 (15.0, 21.8)	28		17.7 (12.1, 27.0)	7	
Neurodevelopmental outcome			0.34			0.15			<0.001 *			<0.001 *
Normal	12.0 (12.3, 13.4)	29		11.5 (10.4, 13.4)	18		10.5 (9.5, 11.2)	19		15.0 (12.5, 26.5)	4	
Anomaly	13.1 (11.0, 14.0)	14		13.3 (10.0, 21.0)	16		15.2 (11.5, 23.2)	21		14.5 (11.3, 17.2)	14	

* significant for *p* < 0.05.
