# Supplementary material for: The Role of Fetal MRI in the Evaluation of Ventriculomegaly: A Scoping Review
Source: Children (Basel). 2026 Jul 22;13(7):971. doi: 10.3390/children13070971 (PMC13406531; doi:10.3390/children13070971)
Supplement: Supplementary file 1 [file children-13-00971-s001.zip › children-4386401-supplementary.pdf]

Search strategy

Developed in consultation with an academic librarian at the Children's Hospital at Westmead and adapted for MEDLINE, Embase, Maternity and Infant Care, and PubMed.

**Population (foetuses with ventriculomegaly)**	**Foetal MRI**	**Investigations<br>Management<br>Outcomes**
**Keywords**		
ventricular adj3 abnormalit*<br>ventriculomegaly<br>f?etus<br>f?etal<br>pregnanc*	in utero<br>magnetic resonance imag*<br>MRI	*n.b. we did not include search terms for these concepts so as not to have too many irrelevant papers.*
**Subject Headings**		
Brain<br>Multiple Abnormalities<br>Fetus<br>Pregnancy	Magnetic Resonance Imaging	*As above.*

Database: Ovid MEDLINE® including Daily update <1996-current>

Search Strategy*:

1 Brain/ (380802)

2 Abnormalities, Multiple/ (24478)

3 1 and 2 (918)

4 (ventricular adj3 abnormalit*).tw,kf. (1652)

5 ventriculomegaly.tw,kf. (2277)

6 Fetus/ or Pregnancy/ (606930)

7 f?etus.tw,kf. (49432)

8 f?etal.tw,kf. (183897)

9 pregnanc*.tw,kf. (332480)

10 in utero.tw,kf. (22477)

11 Magnetic Resonance Imaging/ (453511)

12 (magnetic resonance imag* or MRI).tw,kf. (400716)

13 3 or 4 or 5 (4813)

14 6 or 7 or 8 or 9 or 10 (711119)

15 11 or 12 (587276)

16 13 and 14 and 15 (521)

***Numbers of papers returned at each step of the search are true as of the initial search conducted 24/07/2024.*
